# The Effect of a Short-Term High-Intensity Circuit Training Program on Work Capacity, Body Composition, and Blood Profiles in Sedentary Obese Men: A Pilot Study

**DOI:** 10.1155/2014/191797

**Published:** 2014-02-23

**Authors:** Matthew B. Miller, Gregory E. P. Pearcey, Farrell Cahill, Heather McCarthy, Shane B. D. Stratton, Jennifer C. Noftall, Steven Buckle, Fabien A. Basset, Guang Sun, Duane C. Button

**Affiliations:** ^1^School of Human Kinetics and Recreation, Memorial University of Newfoundland, St. John's, NL, Canada A1C 5S7; ^2^Faculty of Medicine, Memorial University of Newfoundland, St. John's, NL, Canada A1B 3V6

## Abstract

The objective of this study was to determine how a high-intensity circuit-training (HICT) program affects key physiological health markers in sedentary obese men. Eight obese (body fat percentage >26%) males completed a four-week HICT program, consisting of three 30-minute exercise sessions per week, for a total of 6 hours of exercise. Participants' heart rate (HR), blood pressure (BP), rating of perceived exertion, total work (TW), and time to completion were measured each exercise session, body composition was measured before and after HICT, and fasting blood samples were measured before throughout, and after HICT program. Blood sample measurements included total cholesterol, triacylglycerides, high-density lipoprotein cholesterol, low-density lipoprotein cholesterol, glucose, and insulin. Data were analyzed by paired *t*-tests and one-way ANOVA with repeated measures. Statistical significance was set to *P* < 0.05. Data analyses revealed significant (*P* < 0.05) improvements in resting HR (16% decrease), systolic BP (5.5% decrease), TW (50.7%), fat tissue percentage (3.6%), lean muscle tissue percentage (2%), cholesterol (13%), triacylglycerol (37%), and insulin (18%) levels from before to after HICT program. Overall, sedentary obese males experienced a significant improvement in biochemical, physical, and body composition characteristics from a HICT program that was only 6 hours of the total exercise.

## 1. Introduction

Resistance training and aerobic exercise are established approaches to help manage obesity and associated risk factors [1–5]. Both types of exercise have been prescribed to sedentary and obese individuals, and resulted in improved blood pressure (BP), heart rate (HR), body composition, biochemical markers (insulin, glucose, cholesterol, etc.), and strength [6–15]. Combination training (i.e., aerobic and resistance training combined) appears to have a greater effect on BP, arterial stiffness, body composition, and ${\dot{\text{V}}\text{O}}_{\text{2max}}$ than performing either type of exercise independently [13, 14]. Thus, combination training may be a more optimal mode of exercise prescription for the obese population.

One form of combination training is circuit training (CT) which incorporates both multijoint resistance training and callisthenic exercises that keeps the heart rate elevated for the duration of the training session [16]. During CT an individual moves from exercise to exercise as quickly as possible with very little rest, which results in a short duration exercise session. The rest intervals taken during CT are important because HR, BP, and rate pressure product are increased and remain high as the rest intervals between sets and exercises are decreased [17]. A reduced or lack thereof rest period between CT exercises would significantly increase the physiological stress at which an individual exercises while decreasing the overall exercise time.

The effect of CT on various physiological and anthropometric measurements in sedentary middle-aged subjects has been shown to be intensity dependent. Individuals who performed a 12-week high-intensity circuit training (HICT) program had the greatest reductions in body weight, percentage of fat mass, waist circumference, and blood lactate during a submaximal task and greater improvement in strength when compared to individuals who performed endurance training or low-intensity circuit training [18]. Middle-aged obese individuals who performed a 12-week (3, 50 min sessions) HICT program also had greater reductions in fat mass, blood pressure, total cholesterol, low-density lipoprotein cholesterol, high-density lipoprotein cholesterol, triglycerides, and ApoB and increases in high-density lipoprotein cholesterol when compared to individuals who performed endurance training or low-intensity circuit training [16].

It would, therefore, be of great interest to determine the impact of a short duration HICT program on cardiovascular responses, body composition, blood profile, and physical performance. The proposed exercise program design consisting of the combination of resistance and callisthenic training through a short time commitment represents the innovative approach to challenge some of the current exercise paradigm used in primary health care models. In addition, the literature is sparse regarding metabolic change induced by HICT in obese population (see [10, 19, 20] for more details). The purpose of the current pilot study was therefore to determine how a 4-week HICT program would change key physiological health markers in sedentary obese males. The research question poses whether a 4-week HICT program would affect blood profile, resting HR, resting BP, body composition, and physical performance. Part of this data has been presented elsewhere in abstract form [21].

## 2. Methods

### 2.1. Participants

Eight apparently healthy sedentary male participants (34.3 ± 12.1 yrs, 179.1 ± 5.1 cm, 112.4 ± 20.1 kg) participated in the study. Eligibility of participants for the current study was based upon the following inclusion criteria: (1) ≥20 yrs of age, (2) being considered obese by the bray criteria determined from body fat percentage as measured by dual-energy X-ray absorptiometry (DXA) [22] and (3) being healthy, without any serious metabolic, cardiovascular, or endocrine diseases and not taking any prescribed medications. Participants were considered sedentary because they performed only activities of daily living (ADL) and did not engage in any further exercise throughout the week. Participants were verbally informed of all procedures and, if willing to participate, read and signed a written consent form and a Physical Activity Readiness Questionnaire (PAR-Q) [23] prior to participation. The Memorial University of Newfoundland Human Investigation Committee approved this study (Health Research Ethics Authority number 11.319).

### 2.2. Experimental Design

Participants attended an orientation session two days prior to the start of the HICT program. During the orientation session, an investigator instructed the participants how to properly perform all resistance training exercises that were part of the HICT program. The investigator then assisted the participants in finding an 8–12 repetition maximum (i.e., the maximal amount of weight that could be lifted for 8–12 repetitions) for each resistance training exercise. One day prior to the start of the HICT program, blood samples and DXA scan measurements were collected. The next day the participants began the four-week HICT program. For the duration of the four-week HICT program, participants exercised on Monday, Wednesday, and Friday (a total of 12 exercise sessions). One day following the completion of the HICT program (after HICT), blood sample and DXA scan measurements were collected again. Blood samples were collected on Thursday of each week throughout the HICT program. Participants fasted for 12 hours prior to each DXA scan and each blood sample collection. The total time to complete all data collection was approximately 5 weeks (see Table 1 for a schematic of the schedule followed by participants). Throughout the HICT program, participants were asked not to make any change to their diets. This was done in order to determine the effect HICT would have on any measurements independent of any dietary alterations.

## 3. Exercise Protocol

Participants were instructed to follow the Canadian Society for Exercise Physiology preliminary instructions (no eating, drinking caffeine, smoking, or drinking alcohol for 2, 2, 2, or 6 hours, resp.) [23] before each exercise session. Upon arrival to the exercise facility, participants performed a five-minute warm-up consisting of functional body weight exercises using a combination of the exercises completed in the HICT protocol with minimal weight or step-ups and jumping jacks. Following the warm-up the participants completed the HICT protocol which included (1) squat, (2) bench press, (3) partial curl-up, (4) dead lift, (5) burpee, (6) bent over row, and (7) shoulder press (for exercise descriptions see [24]) under the supervision of an investigator. The participants would complete all exercises in order from 1 to 7 and then repeat the process for a total of 3 times (i.e., 3 sets). Verbal encouragement was given to all participants in an attempt to get them to work as hard and fast as possible with as minimal rest as possible between resistance training exercises and each set (i.e., very high work to rest ratio). Thus, the exercise protocol could have been considered a continuous HICT protocol.

The resistance training exercises were modified (easier or harder) throughout the HICT program depending on the capability (i.e., technique or fatigue) of the participant to complete any given exercise. Participants were asked to complete 8–12 repetitions for each resistance training exercise through the greatest range of motion as possible for each repetition. If the participant failed to reach 8 repetitions on a given set, the weight was reduced in the following set. If the participant reached >12 repetitions on a given set the weight was increased in the following set. The partial curl-up and burpee were completed at a varied number of repetitions for each set (i.e., until the participant reached fatigue).

## 4. Measurements

### 4.1. Heart Rate

HR was recorded to determine the training induced acclimation in resting HR over the HICT program and average training intensity (% of age predicted HR max (220-age)) each participant was exercising during each exercise session. A Polar (T-31, PolarElectro, Kempele, Finland) heart rate monitor was used to measure resting HR and HR during each exercise session for each participant. Throughout each exercise session HR was recorded after each set. The mean HR was then calculated for the whole exercise session.

### 4.2. Rating of Perceived Exertion

RPE was recorded as another measure of training intensity. Rating of perceived exertion was measured using Borg's RPE scale [25]. Participants rated their subjective exercise intensity from a scale of 6–20; six being equivalent to complete rest and 20 being equivalent maximum effort. RPE was recorded after each set throughout the exercise session. The mean RPE was then calculated for the whole exercise session.

### 4.3. Time to Completion

The total amount of time it took each participant to complete each exercise session was recorded. Time was recorded as soon as the participant started the exercise session and was stopped as soon as the participant completed the exercise session.

### 4.4. Total Work

TW for each exercise session was computed as the sum of the “weight lifted × number of repetitions” for most of the exercises. Only the squat, bench press, dead lift, bent over row, and shoulder press exercises were used to calculate TW. Since we could not determine the amount of weight lifted for the burpee and partial curl-up these exercises were not included in the TW equation.

### 4.5. Blood Pressure

Resting BP was taken before and after HICT program using an electronic BP cuff (Physio Logic Auto Inflate BP Monitor; AMG Medical Inc., Montreal, QC). BP was recorded to determine training induced acclimation in resting systolic and diastolic BP over the HICT program. Participants were seated with two feet flat on the floor and the arm supported on a table. An appropriate size cuff was chosen and applied firmly to the participants left arm. The lower margin of the cuff was at heart level and two to three cm above the antecubital space.

### 4.6. Anthropometric and Body Composition Measurements

Participants were weighed to the nearest 0.1 kg in standardized clothing (Health O Meter, Bridgeview, IL). Height was measured using a fixed stadiometer (nearest 0.1 cm). Body mass index (kg/m^2^) was calculated as weight in kilograms divided by participants height in meters squared. Body composition measurements were collected utilizing a DXA Lunar Prodigy (GE Medical Systems, Madison, WI). Version 12.2 of the enCORE software package (GE Medical Systems) was used for DXA analysis. DXA can produce an accurate measurement of adipose tissue within the body with a low margin of error. For this reason, DXA is considered to be one of the most accurate measurements of adiposity and is commonly used as a standard compared to less accurate field methods such as BMI. DXA measurements were performed on participants following the removal of all metal accessories, while lying in a supine position [26, 27]. Body composition measurements included lean body mass, percent lean body mass, fat mass, percent body fat (%BF), percent trunk fat (%TF), percent arm fat (%AF), and percent leg fat (%LF). The aforementioned measurements were used to compare changes in body composition from before to after HICT program. Quality assurance was performed on our DXA scanner daily and the typical CV was 1.3% during the study period. All participants in the study were between the ages of 20 and 59  with body fat percentage greater 30% which categorized them all as obese based upon the Bray criteria [22].

### 4.7. Biochemical Measurements

Fasting blood samples were obtained 5 times (see Table 2 for days in which blood was collected before, throughout, and after HICT program) from all participants by a registered nurse after 12 hours of fasting. Blood samples were stored at −80°C for subsequent analyses. The majority of biochemical markers remain stable under these conditions [28]. Blood markers including total cholesterol, triglycerides, high-density lipoprotein cholesterol (HDL), low-density lipoprotein cholesterol (LDL), and glucose were measured with the Lx20 analyzer (Beckman Coulter Inc., Fullerton, CA). Blood insulin was measured by an immunoassay analyzer (Immulite; DPC, Los Angeles, CA). Insulin resistance and Beta cell function were interpreted using the homeostasis model analysis (HOMA), as described by [29]:

(1) HOMA-IR=[Fasting  Insulin (mU/L)×Fasting  Glucose (mmol/L)]22.5  HOMA-β=[20×Fasting  Insulin  (mU/L)Fasting  glucose(mmol/L)−3.5].

### 4.8. Statistical Analysis

All data analyses were conducted using SPSS statistics computing program version SPSS 18.0 (IBM Corporation, Armonk, NY, USA). To determine our sample size a power calculation was performed with a DSS Research statistical power calculator. Based on previous literature [14, 16, 18] a sample size of 8 was sufficient to achieve an alpha level of 0.05 and a power level of 0.80 to minimize the chance of making a type II error. Assumptions of sphericity were tested using Mauchley's test and if violated degrees of freedom were corrected using Greenhouse-Geisser estimates of sphericity. Before and after HICT program measurements for body composition and resting BP data were statistically analyzed using a paired *t*-test. A one-way ANOVA with repeated measures (i.e., time) was performed on dependent variables: resting heart rate (exercise sessions 1–12), RPE (exercise sessions 1–12), TW (exercise sessions 1–12), time to completion (exercise sessions 1–12), and biochemical measures (before weeks 1, 2, and 3 and one day after HICT program). Tukey LSD post hoc test was used to test for significant interactions within the repeated measures ANOVA. Levels of statistical significance for *t*-tests and *F*-ratios were considered statistically significant at the *P* < 0.05 level. Descriptive statistics for all data are reported as group means ± SDs in text, table, and figures.

## 5. Results

### 5.1. Exercise Intensity

**Figure 1:**
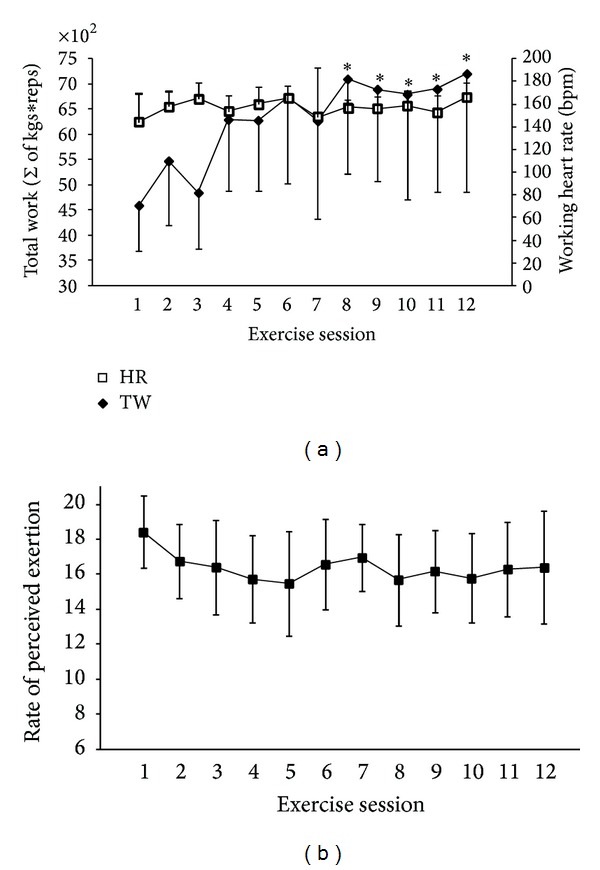
Measurements of exercise intensity. (a) The total work completed (primary *y*-axis) and the working heart rate (secondary *y*-axis). Significant (*P* < 0.05) differences between the first exercise session and exercise sessions 8–12 are indicated by an *. (b) Rate of perceived exertion for each exercise session throughout the HICT program. All data represent means ± SD.

Total work, time to completion, HR, and RPE for each participant were measured during each exercise session. The average TW significantly increased ranging from 50.7% to 53.8% (*P* < 0.04) from exercise session one to exercise sessions 8–12 (Figure 1(a), primary *y*-axis). The time to completion was similar for all participants (~30 minutes) and did not significantly differ between exercise sessions (*P* > 0.5). On average, participants HR was maintained at 85 ± 3.6% of their average age predicted HR_max⁡_ throughout all 12 exercise sessions (Figure 1(a), secondary *y*-axis). Throughout the HICT program, the average RPE was 16.5 ± 2.5; however, there was no significant (*P* > 0.3) difference in participants' RPE for any exercise session (Figure 1(b)).

### 5.2. Resting Heart Rate and Blood Pressure

**Figure 2:**
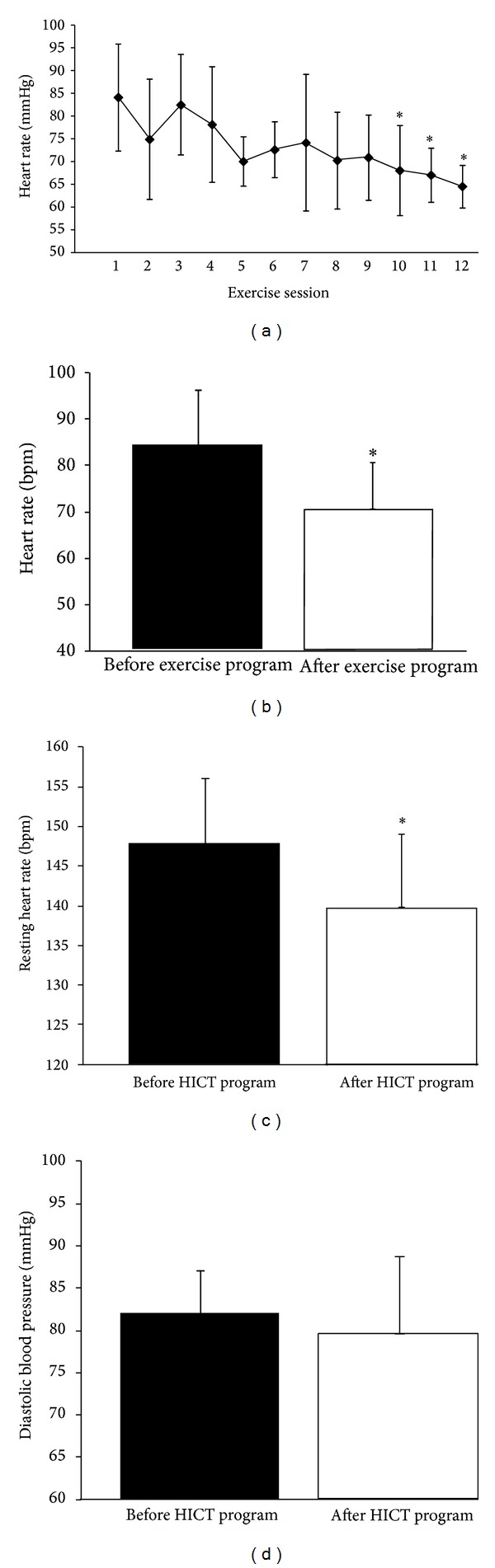
Heart rate and blood pressure measurements. (a) Resting heart rate for each participant measured prior to each exercise session. Significant (*P* < 0.05) differences between the first exercise session and exercise sessions 10–12 are indicated by an *. Differences between before and after HICT program for (b) resting HR, (c) systolic BP, and (d) diastolic BP. Significant (*P* < 0.05) differences are indicated by an * and all data represent means ± SD.

There was a continuous decrease in resting HR from exercise session to exercise session. Although decreases in resting HR were observed from day to day, significant (*P* < 0.05) decreases were not observed until the tenth exercise session compared to the first exercise session (Figure 2(a)). Resting HR decreased by 16.0% (*P* < 0.05) from before to after HICT program (Figure 2(b)). Systolic BP decreased by 5.5% (*P* = 0.03) from before to after HICT program (Figure 2(c)). Diastolic BP decreased by 3.4%, from before to after HICT program; however, this was not significant (Figure 2(d)).

### 5.3. Body Weight and Body Composition

**Figure 3:**
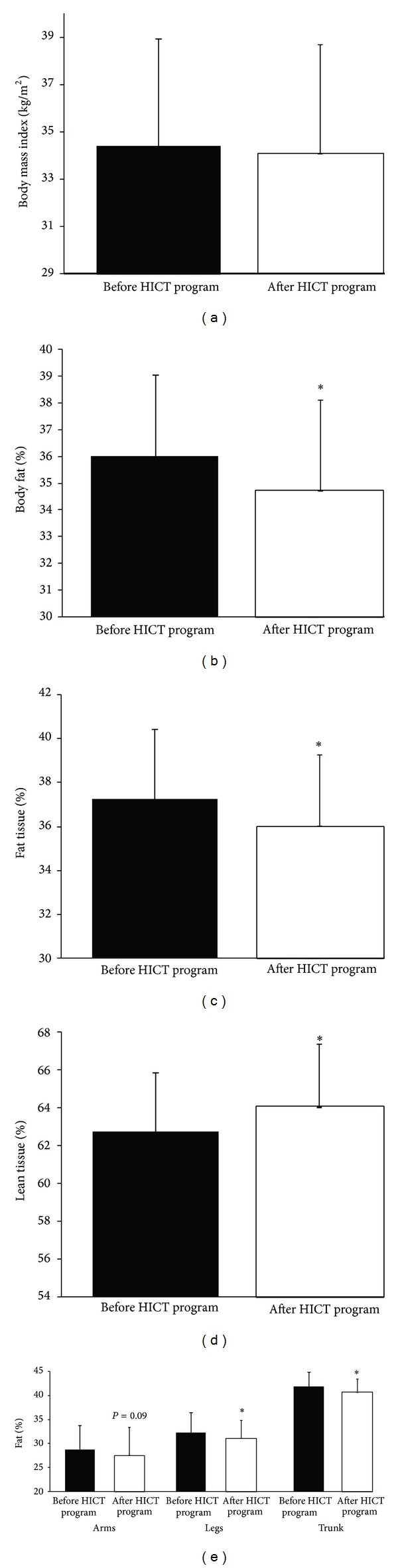
Body composition measurements. Differences between before and after HICT for (a) body mass index, (b) body fat percentage, (c) percentage of body fat tissue, (d) percentage of lean tissue, and (e) percent of fat found in the arm, leg, and trunk. Significant (*P* < 0.05) differences are indicated by an * and all data represent means ± SD.

There were no significant differences in the participant's body mass (112.4 ± 20.2 kg versus 111.1 ± 20.3 kg; *P* = 0.26) or body mass index (*P* = 0.26) (Figure 3(a)) from before to after HICT program. Participants body fat percentage significantly (*P* < 0.01) decreased by 1.6% from before to after HICT program (Figure 3(b)). Lean or fat mass did not change (*P* = 0.26 and *P* = 0.1, resp.) from before to after HICT program. However, when tissue was expressed as a percentage of total mass, the percent fat tissue significantly (*P* < 0.01) decreased (Figure 3(c)) and the percent lean tissue significantly (*P* < 0.01) increased (Figure 3(d)) by 3.6% and 2%, respectively, from before to after HICT program. In addition there was a trend (*P* = 0.09) for a decrease in arm fat percentage of 4.7% and a significant (*P* ≤ 0.01) decrease of 4% and 3% in leg and trunk fat percentages, respectively, from before to after HICT program (Figure 3(e)).

### 5.4. Biochemical Measurements

All raw data for blood sample measurements are reported in Table 2. Fasting total blood cholesterol significantly (*P* ≤ 0.03) decreased by 8.2%, 13%, and 10% at the second, third, and fourth weeks, respectively, of the HICT program compared to baseline (i.e., before HICT). Circulating triacylglycerol significantly (*P* ≤ 0.04) decreased by 30.7%, 34.3%, 36.7%, and 22.4 at the first, second, third, and fourth weeks, respectively, of the HICT program compared to before HICT. There was a trend for insulin (*P* = 0.06), HOMA-IR (*P* = 0.07), and HOMA-*β* (*P* = 0.06) to decrease by 19.1%, 18.9%, and 18.2%, respectively, from baseline to the fourth week of the HICT program. The HICT program had no effect (*P* ≥ 0.2) on fasting blood glucose, HDL cholesterol, and LDL cholesterol.

## 6. Discussion

To the best of the authors' knowledge, this was the first study involving obese males to show that high physiological stress delivered within such a short time window was enough to have an impact on cardiovascular responses, body composition, blood profile, and physical performance. The improvement observed in key health markers induced by our six-hour high-intensity exercise paradigm compares well with studies results using 4 to 8 times longer traditional aerobic, resistance, and circuit training programs (i.e., lasting 12–48 hours).

To monitor exercise intensity and psychophysiological stress during treatment, HR and RPE were measured. The overall participants HR values (see Figure 1(a)) compare well to other HICT studies [16, 18] and attest the high exercise metabolic rate induced by HICT. The 30 min exercise session mean RPE score reached ~16-17 throughout the training program (see Figure 1(b)) providing strong evidence that participants were exercising at a high intensity and were working at the same intensity during each exercise session throughout the HICT program. These outcomes were the result of performing 8–12 repetitions at ~70–80% of their one repetition maximum (1 RM) for every set throughout the HICT program.

The exercise program was designed to maintain repetition and set constant. The significant increase in total work (see Figure 1(a)) was, therefore, mainly due to an increased amount of weight lifted for each repetition (i.e., an increase in strength). A plethora of acute and chronic resistance training studies and reviews report increased strength and muscle mass in a healthy lean population [30–32]. However, obesity research examining the effects of short duration HICT compared to traditional resistance training on work output and/or strength is lacking. For instance, a normal weight sedentary population who performed a long duration HICT program did increase 6 RM leg and bench press performance, compared to the control group [18]. In the same vein, low-volume high-intensity cycling studies have reported chronic muscle metabolic responses comparable to traditional endurance and resistance training [33–35]. In addition, high-intensity exercise provided greater cardiovascular adjustments than lower intensity exercise of the same volume of work [3, 36–38]. Based on the above-mentioned studies, one could have expected no positive effect on strength from such a short duration program; yet, in the current study, the total work (sum of reps × weight) has increased by ~53% from exercise session 1 to exercise session 12 confirming the efficacy of HICT in inducing change in performance (see Figure 1(a)).

Increased BP and resting HR are commonly reported in research on obese individuals and are highly related to obesity-associated risk factors [39, 40]. In the current study six hours of a HICT program sufficed to significantly decrease systolic BP from a hypertensive level (147.9 mmHg) to a high-normotensive level (139.7 mmHg) [2] with no change in diastolic BP and to significantly lower resting HR from 84 bpm to 71 bpm. These results confirmed Paoli et al. [16] who observed a decrease in systolic blood pressure of overweight men using a much longer duration HICT program. Notwithstanding the fact that our exercise program was quite short, the results of the current study compare well with outcomes of chronic population exercise studies using different modes of exercise that were comparable [41] or greater in total exercise duration [4, 38, 42]. The significant decreased resting HR induced by our HICT program, however, contrasts with previous studies reporting no effect of traditional resistance training on this parameter in healthy population. This difference comes from the current HICT program that was designed to elicit high metabolic rate as mirrored by the elevated HR (see Figure 1(a)) during exercise sessions and that has resulted in a decreased resting HR similar to the response triggered by aerobic exercise training [7, 38]. The observed alterations might represent enhanced cardiovascular and haemodynamic responses during exercise and recovery [43] through adjustments in parasympathetic and sympathetic dual talk [44]. Independent of work volume, decreased resting HR and systolic BP were found following traditional resistance training in a cardiovascular disease population [41]. For instance, a recent meta-analysis [45] did report that in chronic and healthy populations high-intensity interval training was superior to induce positive chronic physiological responses ($\dot{\text{V}}$O_2_, BP, HR, lipoproteins, glucose, insulin, cardiac function, oxidative stress, etc.) compared to moderate intensity continuous training. Paoli et al. [16, 18] have also shown that long duration HICT was superior compared to lower intensity circuit training for producing physiological responses similar to those previously listed. One of the relevant findings of the current study attests the efficacy of short duration HICT program in triggering cardiovascular adjustments.

Exercise has lately become of interest among health care professionals. Consequently, exercise physiologists increasingly include efficacy at the center of conceptualization and operationalization of a training program and start challenging exercise program design to optimize time and benefits. Our six-hour HICT program was actually designed to bring about improvement in health markers in a very short time window to comply with the new professional criteria. Although greater reductions in body weight and fat mass and greater increases in lean body mass have been found in longer duration (3, 50 min sessions per week for 12 weeks) HICT studies involving middle-aged obese [16] and sedentary healthy [18] men, our exercise program design has had a significant impact on body composition of our participants. Overall, the participants in the current study gained 1.0 kg of lean mass and lost 1.5 kg of fat mass for a total healthy body composition shift of 2.5 kg and a closer look at the data set revealed significant changes in the fat percentage in the legs and the trunk and a trend towards significant changes in the arms. These results compare well to studies with exercise interventions ranging from 12 to 16 weeks [9, 12, 46] and confirm that HICT is as efficient in reducing body weight and body fat and in increasing lean body mass than low-intensity circuit training and endurance training. Interestingly, both short (the current study) and long duration [16, 18] HICT improved body composition in obese individuals who were not on any dietary restrictions.

In the current study, from the first week (~90 minutes of HICT) and second week (~180 minutes of HICT) and onward, triacylglycerol levels and cholesterol, respectively, decreased and remained decreased throughout the HICT program. Indeed, the reduction in cholesterol and triacylglycerol levels in the current study was of the same magnitude as that reported in the long duration HICT study [16]. Our findings support previous investigations reporting exercise induced decreases in lipids in the absence of dietary restrictions [16, 47, 48]. The results show that only twelve 30 min HICT sessions over a 4-week period can significantly attenuate insulin resistance in nondiabetic adult obese males, a finding that was not replicated in a longer duration low-frequency HICT program for type 2 diabetic patients [49]. Our data, along with others [50, 51], confirms that short duration high-intensity exercise is a time-efficient modality to significantly improve fitness and attenuate hyperglycemia. If the attenuation in insulin resistance is in fact primarily due to local muscle change in response to high-intensity resistance training, then perhaps the larger the number and size of muscles stimulated the greater the improvement in insulin action. Finally, our data demonstrates that HICT induced changes in blood markers (i.e., triacylglycerol, cholesterol, and insulin) are total exercise time dependent and that 6 hours of HICT was not long enough to induce changes in a variety of other blood markers (see Table 2) in obese males.

There were a few limitations in this study that should be considered. The sample population was healthy obese individuals with no comorbidities, which would probably be the one type of “at risk” population that could do the HICT program without problem. The type of training employed here and subsequent results may not be applicable to individuals with one or more chronic diseases. The sample size of the current research is small. However, the complexity of the training program did call for additional caution regarding participant recruitment and we did ensure that only well-motivated persons enrolled in our study, a procedure that has limited the number of participants. Although we did not use the appropriate HR maximum equations for determining exercise intensity in an obese population [52], we found no difference (*P* = 0.9) between HR based exercise intensities by using the age predicted HR maximum and the obesity predicted HR maximum equation.

To reduce biomarker variability, blood samples were collected following 12 hr fasts and at the same time of day for each participant. Therefore, the changes in triacylglycerol levels seen in the current study were probably not due to one day of exercise but rather a cumulative effect of several exercise sessions as no differences were reported in a previous study between one bout of exercise at 24 and 48 hours [53]. Finally, although we did not place any dietary restrictions on the participants our HICT program did bring some change in key health markers. One can expect that combining a HICT program and a proper diet may have enhanced the current health outcomes.

The findings of this pilot study are very promising. We showed that a short duration HICT program could positively improve several physiological health markers in obese males. Most of the improvements are comparable to those found in much longer duration HICT, resistance training, and aerobic exercise programs. Since efficacy became the keyword in exercise programming and a “lack of time” is the most quoted deterrent for participating in an exercise program [3], short duration high-intensity exercise may come to the forefront as the exercise prescription of choice for healthy people and maybe for those who are at risk of chronic disease. However, further research is needed to evaluate the effect of HICT on at risk or chronic diseased populations.

## Figures and Tables

**Table 1:** A five-week schedule for high-intensity circuit training (HICT) program. Dual emission X-ray absorptiometry (DXA).

	Sunday	Monday	Tuesday	Wednesday	Thursday	Friday	Saturday
Week 1				Orientation	Before HICT blood work and DXA scan	Exercise session 1	
Week 2		Exercise session 2		Exercise session 3	Blood work	Exercise session 4	
Week 3		Exercise session 5		Exercise session 6	Blood work	Exercise session 7	
Week 4		Exercise session 8		Exercise session 9	Blood work	Exercise session 10	
Week 5		Exercise session 11		Exercise session 12	After HICT blood work and DXA scan		

Heart rate (HR), beats per minute (BPM), systolic blood pressure (SBP), diastolic blood pressure (DBP), and body mass index (BMI).

**Table 2:** Blood sample measurements from baseline (before HICT program), weeks 1, 2, and 3 of the HICT program, and after the final exercise session in week 4 (after HICT program). Blood sample measurements and *P* values that are bolded indicate that there was a significant (*P* < 0.05) difference from before HICT program measurements. All data represent means ± SD. Low-density lipoprotein (LDL), high-density lipoprotein (HDL), homeostasis model analysis (HOMA), insulin resistance (IR), and beta cell function (*β*).

Blood serum measurements	Baseline	Week 1—HICT	*P* value	Week 2—HICT	*P* value	Week 3—HICT	*P* value	Week 4—HICT	*P* value
Total cholesterol (mmol/L)	4.90 ± 0.60	4.75 ± 0.53	0.08	4.50 ± 0.45	**0.03**	4.26 ± 0.75	**0.03**	4.43 ± 0.63	**0.01**
Triacylglycerol (mmol/L)	2.64 ± 1.94	1.83 ± 1.15	**0.04**	1.71 ± 1.15	**0.00**	1.67 ± 1.09	**0.01**	2.05 ± 1.61	**0.04**
HDL cholesterol (mmol/L)	0.98 ± 0.21	1.04 ± 0.23	0.23	1.00 ± 0.29	0.75	0.96 ± 0.27	0.49	0.96 ± 0.28	0.64
LDL cholesterol (mmol/L)	2.72 ± 0.69	2.88 ± 0.52	0.39	2.72 ± 0.27	0.98	2.55 ± 0.63	0.52	2.53 ± 0.53	0.25
Glucose (mmol/L)	4.90 ± 0.35	5.00 ± 0.41	0.22	5.00 ± 0.50	0.43	4.82 ± 0.38	0.34	4.77 ± 0.35	0.20
Insulin (pmol/L)	71.68 ± 52.37	71.44 ± 55.34	0.67	77.80 ± 51.91	0.92	66.08 ± 62.27	0.29	58.04 ± 59.43	0.06
HOMA-IR	2.17 ± 1.48	2.21 ± 1.65	0.77	2.47 ± 1.53	0.84	2.00 ± 1.85	0.31	1.76 ± 1.83	0.07
HOMA-*β*	177.32 ± 156.28	156.48 ± 132.67	0.40	161.71 ± 132.40	0.72	156.79 ± 154.06	0.22	141.11 ± 133.50	0.06

## References

[1] Church T (2011). Exercise in obesity, metabolic syndrome, and diabetes. *Progress in Cardiovascular Diseases*.

[2] Durstine JL, Moore GE, Painter PL, Roberts SO (2009). *Exercise Management for Persons with Chronic Diseases and Disabilites*.

[3] Gibala MJ, Little JP, Macdonald MJ, Hawley JA (2012). Physiological adaptations to low-volume, high-intensity interval training in health and disease. *Journal of Physiology*.

[4] Jakicic JM, Marcus BH, Gallagher KI, Napolitano M, Lang W (2003). Effect of exercise duration and intensity on weight loss in overweight, sedentary women: a randomized trial. *Journal of the American Medical Association*.

[5] Strasser B, Schobersberger W (2011). Evidence for resistance training as a treatment therapy in obesity.. *Journal of Obesity*.

[6] Alkahtani SA, King NA, Hills AP, Byrne NM (2013). Effect of interval training intensity on fat oxidation, blood lactate and the rate of perceived exertion in obese men. *Springerplus*.

[7] Chaudhary S, Kang MK, Sandhu JS (2010). The effects of aerobic versus resistance training on cardiovascular fitness in obese sedentary volunteers. *Asian Journal of Sports Medicine*.

[8] Cornelissen VA, Fagard RH, Coeckelberghs E, Vanhees L (2011). Impact of resistance training on blood pressure and other cardiovascular risk factors: a meta-analysis of randomized, controlled trials. *Hypertension*.

[9] DeStefano RA, Caprio S, Fahey JT, Tamborlane WV, Goldberg B (2000). Changes in body composition after a 12-wk aerobic exercise program in obese boys. *Pediatric Diabetes*.

[10] Donnelly JE, Blair SN, Jakicic JM, Manore MM, Rankin JW, Smith BK (2009). Appropriate physical activity intervention strategies for weight loss and prevention of weight regain for adults. *Medicine and Science in Sports and Exercise*.

[11] Fett CA, Fett WCR, Marchini JS (2009). Circuit weight training vs jogging in metabolic risk factors of overweight/obese women. *Arquivos Brasileiros de Cardiologia*.

[12] Fleck SJ, Mattie C, Martensen HC (2006). Effect of resistance and aerobic training on regional body composition in previously recreationally trained middle-aged women. *Applied Physiology, Nutrition and Metabolism*.

[13] Ho SS, Dhaliwal SS, Hills AP, Pal S (2012). The effect of 12 weeks of aerobic, resistance or combination exercise training on cardiovascular risk factors in the overweight and obese in a randomized trial. *BMC Public Health*.

[14] Ho SS, Radavelli-Bagatini S, Dhaliwal SS, Hills AP, Pal S (2012). Resistance, aerobic, and combination training on vascular function in overweight and obese adults. *Journal of Clinical Hypertension*.

[15] LaFontaine T (1997). Resistance training for patients with hypertension. *Strength and Conditioning Journal*.

[16] Paoli A, Pacelli QF, Moro T (2013). Effects of high-intensity circuit training, low-intensity circuit training and endurance training on blood pressure and lipoproteins in middle-aged overweight men. *Lipids in Health and Disease*.

[17] Castinheiras-Neto AG, Da Costa-Filho IR, Farinatti PTV (2010). Cardiovascular responses to resistance exercise are affected by workload and intervals between sets. *Arquivos Brasileiros de Cardiologia*.

[18] Paoli A, Pacelli F, Bargossi AM (2010). Effects of three distinct protocols of fitness training on body composition, strength and blood lactate. *Journal of Sports Medicine and Physical Fitness*.

[19] Jakicic JM, Clark K, Coleman E (2001). Appropriate intervention strategies for weight loss and prevention of weight regain for adults. *Medicine and Science in Sports and Exercise*.

[20] McQueen MA (2009). Exercise aspects of obesity treatment. *Ochsner Journal*.

[21] Miller M, McCarthy H, Pearcey GEP (2013). Six hours total of high intensity circuit training improves key physiological health markers in obese males. *Applied Physiology, Nutrition and Metabolism*.

[22] Bray GA (2003). *Contemporary Diagnosis and Management of Obesity and the Metabolic Syndrome*.

[23] (2003). *The Canadian Physical Activity, Fitness & LifeStyle Approach (CPAFLA): CSEP-Health & Fitness Program's Health-Related Appraisal and Counselling Strategy*.

[24] Baechle T, Earle R (2000). *Essentials of Strength Training and Conditioning*.

[25] Borg GAV (1982). Psychophysical bases of perceived exertion. *Medicine and Science in Sports and Exercise*.

[26] Cahill F, Amini P, Wadden D (2013). Short-term overfeeding increases circulating adiponectin independent of obesity status. *PLoS ONE*.

[27] Shea J, Randell E, Vasdev S, Wang PP, Roebothan B, Sun G (2007). Serum retinol-binding protein 4 concentrations in response to shortterm overfeeding in normal-weight, overweight, and obese men. *American Journal of Clinical Nutrition*.

[28] Brinc D, Chan MK, Venner AA (2012). Long-term stability of biochemical markers in pediatric serum specimens stored at-80 °C: a CALIPER Substudy. *Clinical Biochemistry*.

[29] Matthews DR, Hosker JP, Rudenski AS (1985). Homeostasis model assessment: insulin resistance and *β*-cell function from fasting plasma glucose and insulin concentrations in man. *Diabetologia*.

[30] Kraemer WJ, Adams K, Cafarelli E (2002). Progression models in resistance training for healthy adults. *Medicine and Science in Sports and Exercise*.

[31] Kraemer WJ, Ratamess NA (2004). Fundamentals of resistance training: progression and exercise prescription. *Medicine and Science in Sports and Exercise*.

[32] Phillips SM (2000). Short-term training: when do repeated bouts of resistance exercise become training?. *Canadian Journal of Applied Physiology*.

[33] Burgomaster KA, Cermak NM, Phillips SM, Benton CR, Bonen A, Gibala MJ (2007). Divergent response of metabolite transport proteins in human skeletal muscle after sprint interval training and detraining. *American Journal of Physiology*.

[34] Burgomaster KA, Howarth KR, Phillips SM (2008). Similar metabolic adaptations during exercise after low volume sprint interval and traditional endurance training in humans. *Journal of Physiology*.

[35] Gibala MJ, McGee SL, Garnham AP, Howlett KF, Snow RJ, Hargreaves M (2009). Brief intense interval exercise activates AMPK and p38 MAPK signaling and increases the expression of PGC-1*α* in human skeletal muscle. *Journal of Applied Physiology*.

[36] Duncan GE, Anton SD, Sydeman SJ (2005). Prescribing exercise at varied levels of intensity and frequency: a randomized trial. *Archives of Internal Medicine*.

[37] Lee I-M, Paffenbarger RS (2000). Associations of light, moderate, and vigorous intensity physical activity with longevity: the Harvard Alumni Health Study. *American Journal of Epidemiology*.

[38] Nybo L, Sundstrup E, Jakobsen MD (2010). High-intensity training versus traditional exercise interventions for promoting health. *Medicine and Science in Sports and Exercise*.

[39] Hall JE (2000). Pathophysiology of obesity hypertension. *Current Hypertension Reports*.

[40] Re RN (2009). Obesity-related hypertension. *Ochsner Journal*.

[41] Berent R, Von Duvillard SP, Crouse SF, Sinzinger H, Green JS, Schmid P (2011). Resistance training dose response in combined endurance-resistance training in patients with cardiovascular disease: a randomized trial. *Archives of Physical Medicine and Rehabilitation*.

[42] Moraes MR, Bacurau RFP, Casarini DE (2012). Chronic conventional resistance exercise reduces blood pressure in stage 1 hypertensive men. *Journal of Strength and Conditioning Research*.

[43] Fadel PJ, Raven PB (2012). Human investigations into the arterial and cardiopulmonary baroreflexes during exercise. *Experimental Physiology*.

[44] Voulgari C, Pagoni S, Vinik A, Poirier P (2013). Exercise improves cardiac autonomic function in obesity and diabetes. *Metabolism*.

[45] Weston KS, Wisloff U, Coombes JS (2013). High-intensity interval training in patients with lifestyle-induced cardiometabolic disease: a systematic review and meta-analysis. *British Journal of Sports Medicine*.

[46] Treuth MS, Ryan AS, Pratley RE (1994). Effects of strength training on total and regional body composition in older men. *Journal of Applied Physiology*.

[47] Fahlman MM, Boardley D, Lambert CP, Flynn MG (2002). Effects of endurance training and resistance training on plasma lipoprotein profiles in elderly women. *Journals of Gerontology A*.

[48] Fenkci S, Sarsan A, Rota S, Ardic F (2006). Effects of resistance or aerobic exercises on metabolic parameters in obese women who are not on a diet. *Advances in Therapy*.

[49] Hazley L, Ingle L, Tsakirides C, Carroll S, Nagi D (2010). Impact of a short-term, moderate intensity, lower volume circuit resistance training programme on metabolic risk factors in overweight/obese type 2 diabetics. *Research in Sports Medicine*.

[50] Little JP, Gillen JB, Percival ME (2011). Low-volume high-intensity interval training reduces hyperglycemia and increases muscle mitochondrial capacity in patients with type 2 diabetes. *Journal of Applied Physiology*.

[51] Whyte LJ, Ferguson C, Wilson J, Scott RA, Gill JM (2013). Effects of single bout of very high-intensity exercise on metabolic health biomarkers in overweight/obese sedentary men. *Metabolism*.

[52] Miller WC, Wallace JP, Eggert KE (1993). Predicting max HR and the HR-VO2 relationship for exercise prescription in obesity. *Medicine and Science in Sports and Exercise*.

[53] Weise SD, Grandjean PW, Rohack JJ, Womack JW, Crouse SF (2005). Acute changes in blood lipids and enzymes in postmenopausal women after exercise. *Journal of Applied Physiology*.

